# Arsenite Regulates Prolongation of Glycan Residues of Membrane Glycoprotein: A Pivotal Study via Wax Physisorption Kinetics and FTIR Imaging

**DOI:** 10.3390/ijms17030427

**Published:** 2016-03-22

**Authors:** Chih-Hung Lee, Chia-Yen Hsu, Pei-Yu Huang, Ching-Iue Chen, Yao-Chang Lee, Hsin-Su Yu

**Affiliations:** 1 Department of Dermatology, Kaohsiung Chang Gung Memorial Hospital and Chang Gung University College of Medicine, Kaohsiung 83301, Taiwan; dermlee@gmail.com; 2 Biomedical and Molecular Imaging Lab, X-ray and IR Imaging Group, National Synchrotron Radiation Research Center, 101 Hsin-Ann Road, Hsinchu Science Park, Hsinchu 30076, Taiwan; hsu.cy@nsrrc.org.tw (C.-Y.H.); pyhuang@nsrrc.org.tw (P.-Y.H.); 3 Beamline Group, National Synchrotron Radiation Research Center, Hsinchu 30076, Taiwan; cqc@nsrrc.org.tw; 4 Department of Optics and Photonics, National Central University, Chung-Li 32001, Taiwan; 5 National Institute of Environmental Health Sciences, National Health Research Institutes, No. 35, Keyan Road, Zhunan Town, Zhunan 35053, Taiwan; 6 Department of Dermatology, Kaohsiung Medical University, Kaohsiung, No. 100, Shih-Chuan 1st Road, Kaohsiung 807, Taiwan

**Keywords:** wax physisorption kinetics, synchrotron-radiation-based FTIR, focal-plane-array-based FTIR, glycosylation

## Abstract

Arsenic exposure results in several human cancers, including those of the skin, lung, and bladder. As skin cancers are the most common form, epidermal keratinocytes (KC) are the main target of arsenic exposure. The mechanisms by which arsenic induces carcinogenesis remains unclear, but aberrant cell proliferation and dysregulated energy homeostasis play a significant role. Protein glycosylation is involved in many key physiological processes, including cell proliferation and differentiation. To evaluate whether arsenite exposure affected protein glycosylation, the alteration of chain length of glycan residues in arsenite treated skin cells was estimated. Herein we demonstrated that the protein glycosylation was adenosine triphosphate (ATP)-dependent and regulated by arsenite exposure by using Fourier transform infrared (FTIR) reflectance spectroscopy, synchrotron-radiation-based FTIR (SR-FTIR) microspectroscopy, and wax physisorption kinetics coupled with focal-plane-array-based FTIR (WPK-FPA-FTIR) imaging. We were able to estimate the relative length of surface protein-linked glycan residues on arsenite-treated skin cells, including primary KC and two skin cancer cell lines, HSC-1 and HaCaT cells. Differential physisorption of wax adsorbents adhered to long-chain (elongated type) and short-chain (regular type) glycan residues of glycoprotein of skin cell samples treated with various concentration of arsenite was measured. The physisorption ratio of beeswax remain/n-pentacosane remain for KC cells was increased during arsenite exposure. Interestingly, this increase was reversed after oligomycin (an ATP synthase inhibitor) pretreatment, suggesting the chain length of protein-linked glycan residues is likely ATP-dependent. This is the first study to demonstrate the elongation and termination of surface protein-linked glycan residues using WPK-FPA-FTIR imaging in eukaryotes. Herein the result may provide a scientific basis to target surface protein-linked glycan residues in the process of arsenic carcinogenesis.

## 1. Introduction

Chronic arsenic exposure leads to several human cancers, including skin cancer, lung cancer, bladder cancer, and liver cancer [1,2,3]. Arsenic has been declared a Class I carcinogen by the International Agency for Research on Cancer (IARC). Skin cancers are the most common cancers caused by arsenic exposure and usually herald other arsenic cancers [4]. To reduce the risk of arsenic-induced adverse health effects, understanding the pathophysiology of arsenical skin cancers may help develop clinic treatments to halt the chronological march of the arsenic cancers. The molecular mechanisms of arsenic carcinogenesis still remain unclear, although aberrant cell proliferation, increased oxidative stresses, chromosome abnormalities and immune dysregulation might be involved [5,6,7,8]. Aberrant cell proliferation followed by uncontrolled growth are the critical events in the initiation of carcinogenesis. Arsenic exposure induces aberrant cell proliferation was observed in several kinds of human cells, including human epidermal KC, cardiac progenitor cells, prostate epithelial cells, and lung epithelial cell [9,10,11,12]. In epidermal KCs, we reported that the exposure of low doses of sodium arsenite (≤1 µM) induce aberrant cell proliferation through mitochondrial biogenesis by upregulation of mitochondrial transcription factor A (mtTFA) [13].

Glycosylation of protein is the most common post-translational modification and it is involved in many crucial physiological processes, including protein folding and unfolding, cell–cell and cell–matrix interactions, and cellular differentiation [14]. Aberrant protein glycosylation was strongly suggested to relate to incomplete synthesis and neo-synthesis of protein glycosylation, which would induce more population of the branched or elongated protein-linked glycan residues of glycoprotein during protein glycosylation within cell [15,16,17,18]. The alteration of structures of protein-linked glycan residues was proven during the progression of carcinogenesis and the exposure of arsenic trioxide (6 µM) for glioma cell lines (SHG44, U87 and U251) [19]. Therefore, in the arsenic-targeting cells, including epidermal KCs, arsenic exposure might play a key role to induce aberrant glycosylation that could alter the chain length and structure of protein-linked glycan residues, leading to abnormal cell proliferation.

The state-of-the-art FTIR imaging is a non-invasive and non-destructive technique which could provide the laterally-resolved infrared spectra in the interest area of samples [20,21,22]. During the past decade, FTIR imaging has been utilizing for differentiating malignancy from normal cells or tissue based on the spectral marker of cancers [23,24,25]. The alteration of membrane protein-linked glycan residues of oral cavity cancer and ovarian cancer was investigated using WPK-FPA-FTIR imaging, and diagnostic wax adsorbents, n-pentacosane (n-C_25_H_52_) and beeswax (C_30_H_61_CO_2_C_15_H_31_) were employed for targeting short-chain (regular type) and long-chain (elongated type) protein-linked glycan residues, respectively. WPK was employed to investigate the physisorption strength caused by van der Waals interaction between wax adsorbent molecules and glycan residues of membrane glycoprotein of waxed cell samples. The physisorption strength can be correlated with the differential infrared (IR) absorbance of characteristic IR absorption in the mid-infrared range of 3000–2800 cm^−1^ between wax adsorbent remain adhered onto the waxed sample surface after dewaxing for certain period of time and the sample before waxing [26,27]. In this study, we aimed to investigate the correlation between the chain length alteration of glycan residues of membrane glycoproteins on cell surface and cell proliferation of KC, HaCaT and HSC1 cells during the arsenite exposure by using the technologies of WPK-FPA-FTIR imaging. Furthermore, abnormal glycosylation processes of glycoprotein may require secured energy supply from mitochondria during arsenite-induced cell proliferation. We then asked whether the glycan residues structure of glycoprotein would be mediated by ATP. The ATP synthesis pathway in arsenite-treated cells was blocked by pre-treatment and co-treatment with oligomycin, an ATP synthase inhibitor, followed by WPK-FPA-FTIR imaging.

## 2. Result

### 2.1. FTIR Analysis of Skin Cells Exposed to Oligomycin and Sodium Arsenite

**Figure 1:**
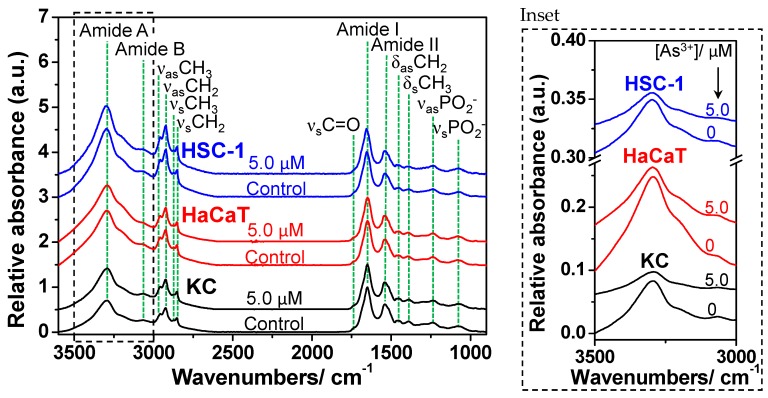
The representative normalized FTIR reflectance spectra of cell samples on low-e slides after arsenite exposure by co-cultured with sodium arsenite (0 and 5 μM) in the range of 3600–900 cm^−1^; the inset plot showed the amide A band of cell samples without spectral normalization in the range of 3500–3000 cm^−1^. All FTIR reflectance spectra were acquired by using a FTIR spectrometer coupled with an infrared integrating sphere.

**Figure 2:**
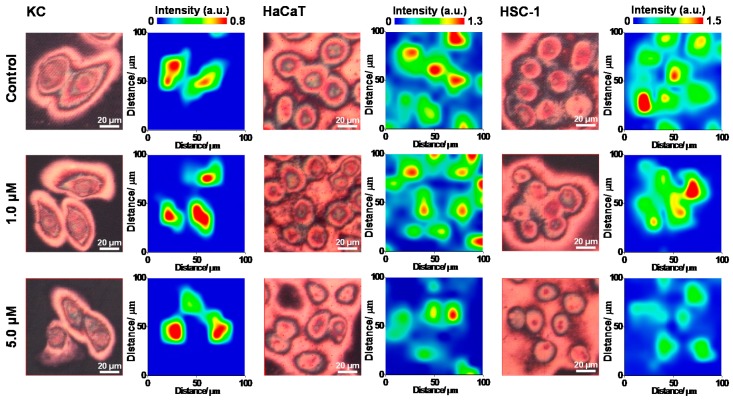
Peak height images of amide A band for KC, HaCaT and HSC-1 cell samples in the field of view of 100 × 100 µm^2^ were presented after treating with sodium arsenite using SR-FTIR microspectroscopy. The FTIR images of peak height of amide A were collected at 3294 cm^−1^ for cell samples (representative data were shown; 3 repeated experiments, 3 replicates, at least 10 cells were measured).

The normalized FTIR reflectance spectra of KC, HaCaT and HSC-1 cells showed the obvious intensity differences of the amide A band for cell samples after treating with different doses of sodium arsenite as compared with the corresponding cell sample without arsenite exposure (Figure 1); however, the cell samples exhibited rare spectral alteration after oligomycin pretreatment at the dose of 0.5 µg/mL (data not shown), and the IR absorption bands of cell samples were assigned as exhibited in Table 1. Secondary structure alteration of protein has been found in an ultrahigh dose of arsenite exposure (100 µM) with human acute promyelocytic leukemia cells [28]. Herein the intensity of amide A was decreased after arsenite exposure at 5 µM (Figure 1), of which the concentration was reported to be harmful to cell survival [13], even in the samples pretreated with oligomycin (data not shown), suggesting the decrease of absorbance of amide A was dominated by sodium arsenite. The intensity of amide A was sensitively altered for skin cell samples after arsenite exposure; therefore, the amide A could be a spectral signpost for cell proliferation during arsenite exposure. Furthermore, the result of peak height images of amide A using SR-FTIR microspectroscopy showed that the protein content of KC and HSC-1 cells was increased after exposure to sodium arsenite at 1 µM. On the other hand, an obvious decrease was found after exposure to sodium arsenite at 5 µM (Figure 2). However, HaCaT cells exhibited a downward trend with increasing arsenite concentration. According to the result of decreasing peak height of the amide A band after arsenite exposure in all three cells treated with higher dose of arsenite, we strongly suggested that three skin cells be obviously stressed herein during the exposure of sodium arsenite of 5 µM as compared with corresponding control cell samples (Figure 1).

### 2.2. Alteration of Surface Protein-Linked Glycan Residues Induced by Arsenite Exposure

**Figure 3:**
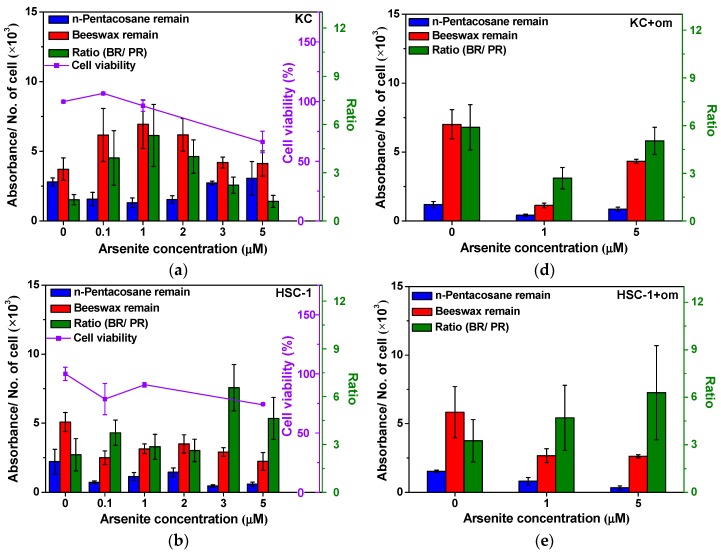

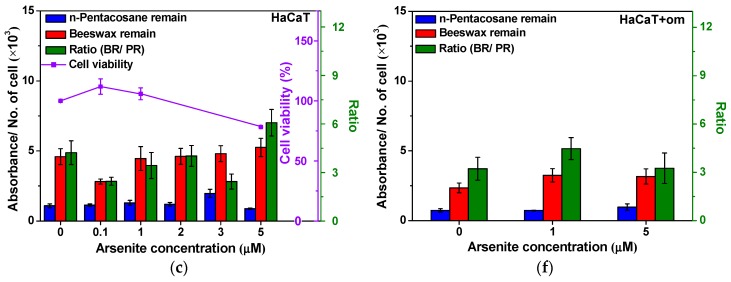
A representative profile of average amount of wax adsorbent remaining adhered on the cell surface of a single cell (blue and red column, left axis) acquired by WPK-FPA-FTIR imaging and cell viabilities (purple line, right axis with an inner ticks) after treating with a variety of concentrations of sodium arsenite and co-cultured with oligomycin (om) and sodium arsenite. (**a**,**d**) KC; (**b**,**e**) HSC-1; and (**c**,**f**) HaCaT cells. The glycan residues population ratio (long-chain/short-chain; green column, right axis with an outer ticks) was used to estimate the ratio of beeswax remaining/n-pentacosane remaining (BR/PR) in different cultured conditions.

A biphasic dose-response of cell proliferation was observed on KC and HaCaT cells after arsenite exposure to skin cells (Figure 3a,b), which was consistent with previous studies [13,29]. Therefore, we suggested that the treating dose of 1 μM sodium arsenite to KC and HaCaT cells should be a critical point for promoting cell proliferation or enhancing cell apoptosis. Nevertheless, higher doses led to a poor cell viability when cells were treated with a dose higher than 1 μM sodium arsenite. The arsenic-induced cell proliferation of skin cancer cells herein was consistent with other literature [6,30]. Moreover, protein glycosylation is involved in many key physiological processes, including cell proliferation and differentiation; therefore, the aberrant glycosylation of surface protein-linked glycan residues of skin cells might play an important role during cellular differentiation as regulated by arsenic [14]. WPK-FPA-FTIR imaging was employed to investigate the alteration of surface protein-linked glycan residues of membrane glycoprotein of cell samples after arsenite exposure by estimating the amount of wax adsorbent remaining on the cell sample based on the IR absorbance alteration of characteristic IR absorption of wax adsorbent in the mid-infrared spectra. The total estimated amount of wax adsorbents (n-pentacosane or beeswax) remaining on the cell surface of the waxed skin cell sample after xylene washing for 6 s was correlated to the differential IR absorbance of wax adsorbent by subtracting the IR absorbance of the same cell sample before waxing in the mid-infrared range of 3000–2800 cm^−1^ for all cells in the detection area of 100 × 100 µm^2^. However, the value of total estimated amount of wax adsorbent remaining on the cell sample is proportional to the number of cells in the detection area of the cell sample. Therefore, averaged estimated amount of wax adsorbent remaining on a single cell was used herein by dividing the total estimated amount of wax adsorbent by the number of cells in the detecting area corresponding to the averaged population of regular or elongated glycan residues on the cell surface of a single cell.

**Figure 4:**
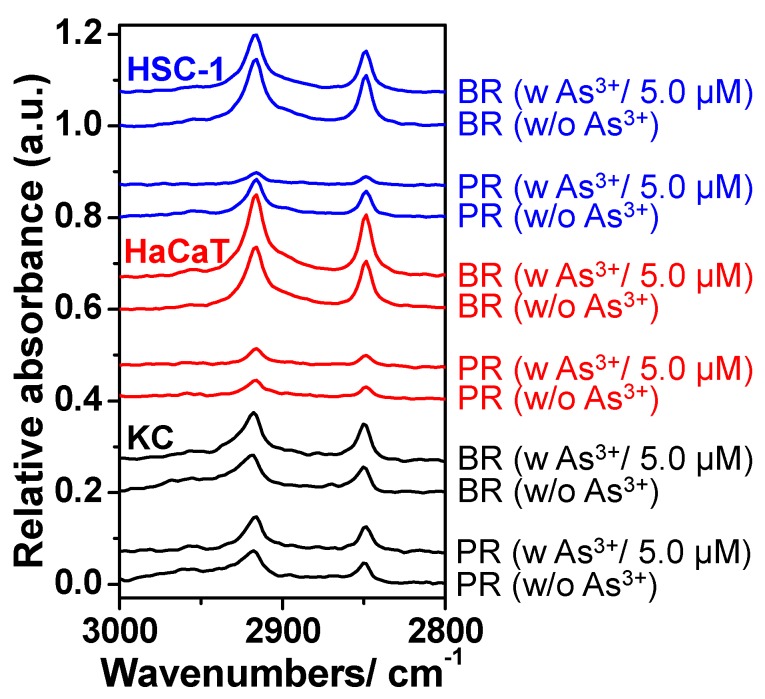
Characteristic IR absorbance of n-pentacosane remaining (PR) and beeswax remaining (BR) adhered on cell samples with (*w*) and without (*w*/*o*) sodium arsenite (As^3+^) exposure after a dewaxing procedure via xylene washing, using the WPK-FPA-FTIR imaging technique. The IR absorbance spectrum data was determined relative to that of the corresponding non-waxed cell sample.

All waxed cell samples after dewaxing procedure by xylene washing actually exhibited more intense characteristic IR absorbance of beeswax remaining adhered to the cell surface than that of n-pentacosane remaining after exposure to 5 µM arsenite, indicating the distinguishable physisorption of both wax adsorbents as compared with the same cell samples before waxing (Figure 4). For HSC-1 cells, there was a slight decrease for both beeswax and n-pentacosane remaining in waxed HSC-1 cells after exposure to 5 µM arsenite. For HaCaT cells, the beeswax remaining of waxed HaCaT cells exhibited a slightly increasing trend as comparing with waxed samples without arsenite exposure. Based on these findings, arsenite exposure might interfere the expression of glycan residues of membrane glycoproteins in cancer cells (HSC-1 and HaCaT cells).

In terms of the association of the wax remains and the cell viabilities, the result of WPK-FPA-FTIR imaging of skin cells elucidated that the profile of beeswax remaining adhered on the cell surface of KC and HSC-1 cells was positively correlated with their cell viability (Figure 3a,b). HaCaT cells showed a nearly constant amount of beeswax remaining on the cell surface during arsenite exposure but a significant drop in what remained after treating with 0.1 μM sodium arsenite (Figure 3c). Furthermore, the population ratio of long-chain to short-chain protein-linked glycan residues of membrane glycoprotein, which was employed corresponding to the ratio of beeswax remain/n-pentacosane remain, in KC and HSC-1 cells was increased during the arsenite exposure, indicating long-chain protein-linked glycan residues dominant within incubation of 24 h.

### 2.3. The Alteration of Surface Protein-Linked Glycan Residues of Skin Cells Treated with Oligomycin and Sodium Arsenite

To further investigate the association between the structure alteration of protein-linked glycan residues with ATP, skin cells were pretreated with oligomycin, an ATP synthase inhibitor, before arsenite exposure. Residues of n-pentacosane and beeswax exhibited a downhill trend after treating KC and HSC-1 cells with oligomycin and arsenite exposure (Figure 3d,e); however, HaCaT cells displayed an increasing trend (Figure 3f). Herein oligomycin rarely affected the population of short-chain protein-linked glycan residues of cell surface of skin cells treated with sodium arsenite but long-chain protein-linked glycan residues were obviously altered by arsenite to KC and HSC-1 cells as compared with corresponding control cell samples. Of note, HaCaT cells showed rare alterations for both types of glycan residues after treating with oligomycin and sodium arsenite, suggesting the elongation of protein-linked glycan residue might depend on ATP in KC and HSC-1 cells but not in HaCaT cells. An increased ratio of beeswax remaining/n-pentacosane remaining ratio (BR/PR) was observed for KC (Figure 3a,d) and HSC-1 cell (Figure 3b,e) samples without arsenite exposure after oligomycin pretreatment; nevertheless, the BR/PR ratio of HaCaT cell samples was decreased (Figure 3c,f). These results suggested that ATP-related processes might be involved in the glycosylation of membrane glycoprotein, and the interference of ATP synthesis might alter the expressions of elongated type and regular type glycan residues on the membrane glycoproteins.

## 3. Discussion

To investigate the mechanism of arsenite-induced carcinogenesis, normal human keratinocyte, transformed or cancerous skin cells are often utilized. Aberrant cell proliferation by arsenic may result from various biological mechanisms [13,31,32,33]. In addition, abnormal protein glycosylation may also lead to aberrant cell proliferation. The long-chain protein-linked glycan residues exhibited a strong correlation with cell viability of KC and HSC-1 cells herein during arsenite exposure according to the results of WPK-FPA-FTIR imaging for skin cells. The high dose of arsenite exposure was also reported to induce structural changes in immature lipid-linked glycoprotein [34] and high-mannose N-linked glycans [35]. Furthermore, the glycosylation of N-linked glycans was highly regulated by the availability of intracellular *N*-acetylglucosamine [36] and would have a strong interaction with sodium arsenite for rendering *E. coli* against arsenite toxicity [37]. Taken together, the arsenite-induced aberrant cell proliferation may result from the pathway of abnormal protein glycosylation.

Based on previous studies of bacteria, the carbonhydrate component of the cell wall was demonstated to strongly interact with arsenite [38]; furthermore, the assembly of lipopolysaccharide O9a antigen in *E. coli* was also confirmed to be ATP-dependent [39]. A subsequent study validated that chain termination reaction in biosynthesis of the *E. coli* O9a *O*-polysaccharide is regulated by the chain-length regulator, WbdD, which catalyzes the addition of methyl phosphate to the non-reducing terminus of the growing glycan [40]. Consequently, ATP would be involved in the pathway for glycosylation of protein. In arterial smooth muscle cells, arsenite inhibited general proteoglycan synthesis, which might play a role of bias in the progression of atherosclerosis and vascular diseases [41]. In addition, this process is protected by the pretreatment of bismuth [42]. In the current study, we demonstrated that the relative length of protein-linked glycan residues may be dependent on the ATP in the model of arsenite-treated primary KC. This is a novel study to demonstrate the dependency of prolongation and termination of protein-linked glycan residues on ATP in eukaryotes by incorporating SR-FTIR microspectroscopy and WPK-FPA-FTIR imaging.

This study has some limitations. Firstly, for the sample cells without clear cell morphology or margin, it would not be easy to estimate the residual wax adsorbent remaining on the sample at the same defined position. Secondly, the imaged area may be limited to the center of slide, though deposits of wax adsorbent around the edge of the slide might be different. Thirdly, oligomycin, an ATP synthase inhibitor, might regulate the elongation of protein-linked glycan residues of membrane glycoprotein. It may be possible that oligomycin has effects other than on ATP. Therefore, further specific ATP blockings herein might help delineate this issue unsettled. Nevertheless, this pivotal study demonstrated that the WPK-FPA-FTIR imaging technique was employed to estimate the amount of different chain lengths of glycan residues of membrane glycoprotein using two different aliphatic length of wax adsorbents (n-pentacosane and beeswax) according to their IR absorbance alteration in the spectral range of 3000–2800 cm^−1^. Hence, different aliphatic lengths of wax adsorbent could also be utilized to acquire useful information about the glycosylation alteration of membrane glycoprotein.

## 4. Methods

### 4.1. Cell Culture and Cell Sample Preparation

Human KC were cultured herein and obtained from adult foreskins through routine circumcision as we previously described [43]. HaCaT and HSC-1 cell lines were human squamous cell carcinoma cell lines and obtained from ATCC and National Institute of Biomedical Innovation in Japan, respectively. Cell samples were cultivated on MirrIR low-e slides (Kevley Technologies, Chesterland, OH, USA) in the absence or presence of indicated sodium arsenite (0–5 µM) for 24 h at 37 °C incubation supplied with 5% CO_2_. Moreover, to investigate the association between mitochondrial function and the alteration of the protein-linked glycan residue structure during arsenite exposure for skin cells, an ATP synthase inhibitor, oligomycin, in oxidative phosphorylation which responds to oxidative phosphorylation of adenosine diphosphate (ADP) to ATP was used to pretreat cells. Cell samples were treated with 0.5 µg/mL oligomycin for 1 h prior to 24 h of sodium arsenite exposure. For FTIR spectroscopic measurement, cell samples were fixed with 4% formaldehyde on MirrIR low-e microscope and then washed twice with PBS and deionized water, respectively.

Cells were seeded in 6-well plates at 5.5 × 10^4^ cells/well. Cells were treated with 0.5 µg/mL oligomycin (Sigma-Aldrich., St. Louis, MO, USA) for 1 h prior to arsenite exposure using sodium arsenite (Fluka, Buchs, Switzerland) from 0.1 to 5 µM for 24 h, washed with 2 mL ice-cold phosphate buffered saline (PBS), dislodged with trypsin, and pelleted by centrifugation for 5 min at 1200 rpm. Total attached cells were counted using a Neubauer’s chamber (Sigma-Aldrich., St. Louis, MO, USA).

### 4.2. Integrating Sphere for FTIR Reflectance Spectroscopy and SR- FTIR Microspectroscopy

FTIR reflectance spectra of cell samples after arsenite and oligomycin treatments were measured in the mid-infrared range of 3600–900 cm^−1^ using a FTIR spectrometer coupled with an integrating sphere (Mid-IR IntegratIR™, PIKE, Madison, WI, USA). SR-FTIR spectra of cells samples were accumulated 128 scans with resolution of 4 cm^−1^ in the spectral range of 4000–650 cm^−1^ by using the end station of SR-FTIR microspectroscopy, including an FTIR spectrometer (Nicolet 6700, Thermo-Fisher-Nicolet Instruments, Madison, WI, USA) equipped with an IR confocal microscope (Continuµm; Spectra Tech Inc., Oak Ridge, TN, USA) coupled with a single-pixel 50 × 50 µm^2^ liquid nitrogen-cooled (LN-cooled) Mercury-Cadmium-Telluride (MCT) detector and the collimated infrared synchrotron radiation as infrared light source at BL14A1 of National Synchrotron Radiation Research Center (NSRRC) in Taiwan. The third-generation synchrotron of NSRRC is the operation of top-up mode at 1.5-GeV, 360 mA and a period of electron injection of 60 s. In this study, FTIR reflectance spectra and SR-FTIR spectra of cell samples were normalized at amide I intensity and baseline corrected by using OMNIC™ (OMNIC 9.2, Thermo Fisher Scientific, Waltham, MA, USA). The SR-FTIR images of peak height of amide A band of cell samples herein were constructed by collecting the peak height at 3294 cm^−1^ for each lateral mapping point on cell samples, which were irradiated by a focused infrared beam of 10 × 10 µm^2^ by a 32 × Cassegrain objective and scanned on a program-controlled sample stage of IR confocal microscope with step size of 10 µm for three different areas of cell sample on a low-e slide.

The optical path of the end station of SR-FTIR microspectroscopy was continuously purged by dry nitrogen from LN Dewar (XL-100, TAYLOR-WHARTON, Theodore, AL, USA) and an automatic atmospheric suppression function was applied in OMNIC™ for minimizing the rovibration of CO_2_ and water vapor during FTIR data acquisition. The flow rate of dry nitrogen gas was regulated by a gas regulator and a mass flow meter (FMA-1611A-I, OMEGA, Stamford, CT, USA) at 170–180 SLPM after gas regulator for purging the optical path of two sets of FTIR end-stations, for which the relative humidity was maintained at ~7% in the sample compartment of FTIR spectrometer and the sample detection area of the IR microscope at room temperature (around 25 degrees Celsius) in the air-conditioned IR hutch of the BL14A1 infrared beamline at NSRRC.

### 4.3. WPK Procedures and FPA-FTIR Imaging

**Figure 5:**
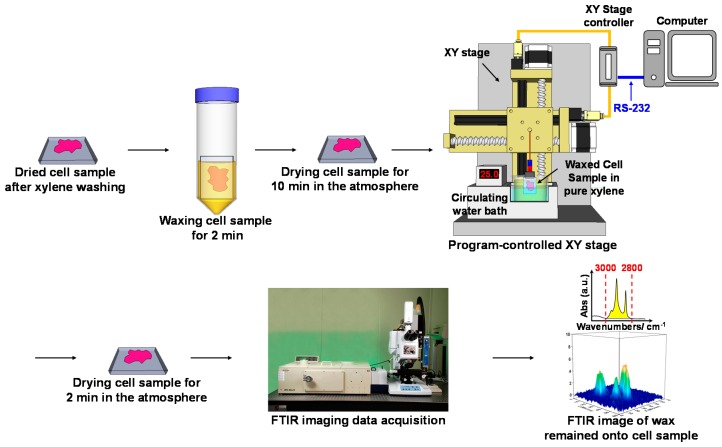
The schematic WPK-FPA-FTIR imaging procedure was demonstrated for estimating the amount of wax adsorbent remaining on the cell sample surface using IR absorbance of wax adsorbent in the range of 3000–2800 cm^−1^.

We developed the WPK to investigate the physisorption strength of van der Waals interaction between wax adsorbent and surface protein-linked glycan residues of waxed cell samples by measuring the differential absorbance of characteristic IR absorption of wax adsorbent remaining on the cell surface, during the period for dewaxing the waxed cell sample by xylene washing at 25 °C as compared with the cell sample before waxing [26,27]. The estimated amount of wax adsorbent remaining on the cell surface was measured at the same certain time period of xylene washing for the waxed cell sample as compared with the cell sample before waxing. This was based on the IR absorbance difference of wax adsorbent between waxed and non-waxed cell samples in the mid-infrared spectral range of 3000–2800 cm^−1^. Based on the neo-synthesis of glycosylation of glycoprotein during cell proliferation of cancer development, elongated glycan residues of glycoprotein have been reported in a variety of cancers [15,16,17,18]; therefore, two wax adsorbents with different length of n-aliphatic chain, n-pentacosane and beeswax, were employed herein as wax adsorbents to adsorb regular and elongated glycan residue of membrane glycoprotein, respectively. Based on the point of view of the rule of “like dissolves like”, stronger interaction is established among similar molecules. Consequently, the more the amount of wax remaining on the cell sample after xylene washing for the same time period as for the waxed cell sample, the greater physisorption might be established between wax adsorbent and cell surface, by contrast. The experimental procedure of WPK for estimating the amount of wax adsorbent remaining on the surface of cell samples using FPA-FTIR imaging was schematically described in Figure 5. A formalin-fixed cell sample was soaked in a glass pool containing 20 mL xylene in a water bath (BL-710D, Yihder, Xinbei, Taiwan) at 55 °C for 10 min to remove the organic contamination during the period of cell sample preparation. Dried cell samples were waxed by soaking in a bath of wax-xylene solution of 20 mL, containing 5.5 wt % of n-pentacosane or beeswax, for 2 min and then dried at room temperature for 10 min in the atmosphere. The period of time for dewaxing the waxed cell sample was precisely preceded by a dewaxing system, a program-controlled XY-stage (ST9090-150, TANLIAN, Taoyuan, Taiwan) remotely controlled via an RS-232 interface on a personal computer (Figure 5). During the dewaxing of the waxed cell sample, the waxed sample was soaked in a glass trough filled with pure xylene of 40 mL by a circulating bath (WB211, P&C Biotech, Hsinchu, Taiwan) at 25 °C for 6 s for the cell samples without oligomycin pretreatment or for 7 s for oligomycin-pretreated cell samples to differentiate the physisorption strength between the arsenite-treated and non-arsenite-treated cell samples.

FPA-FTIR imaging system, including a FTIR spectrometer (IFS 66 v/S, Bruker, Ettlingen, Germany) equipped with an IR microscope (Hypersion 3000, Bruker, Ettlingen, Germany) fitted a LN-cooled 64 × 64 pixels MCT FPA detector (Santa Barbara Focalplane, Goleta, CA, USA), was employed to acquire FTIR spectral images of wax adsorbent remaining on cell samples, and the field of view of 100 × 100 µm^2^ was imaged by using a 15 × Cassegrain objective of IR microscope [44,45]. Each pixel of the MCT FPA detector was utilized for collecting the time-domain spectrum of each corresponding spatial part of cell sample, which were irradiated by infrared radiation modulated by a Michelson interferometer of FTIR, and the FTIR spectrum was produced after transforming the time-domain spectrum by using a fast Fourier transform (FFT) [46]. Herein the FPA-FTIR spectral images of wax adsorbent remaining on the cell sample surface after dewaxing the waxed cell sample for an optimal period of time was constructed by using IR absorbance that integrated the characteristic absorption in the range of 3000–2800 cm^−1^ of FTIR spectrum of cell samples. To depict the relative estimated amount of short-chain and long-chain glycan residues of glycoprotein remaining on the surface of a single cell, the IR absorbance of cell samples was divided by the cell numbers in the field of view of detection area. In this study, FTIR spectra of cell samples were accumulated in 64 scans with a resolution of 4 cm^−1^ in the spectral range of 3600–900 cm^−1^. The optical path of FPA-FTIR end-station imaging was also continuously purged by dry nitrogen in the IR hutch of BL14A1 at NSRRC. The estimated amount of wax adsorbent remaining on the cell sample surface was estimated by using FPA-FTIR imaging based on the absorbance in the mid-infrared range of 3000–2800 cm^−1^, the characteristic IR absorption for wax adsorbent of n-pentacosane and beeswax in this study.

## 5. Conclusions

Abnormal protein glycosylation could regulate either cell proliferation or cell apoptosis. The WPK-FPA-FTIR imaging technique showed that the alteration of chain length of protein-linked glycan residues of membrane glycoprotein was associated with cell proliferation by arsenite exposure in elongation of protein-linked glycan residues on keratinocyte surfaces. Therefore, the alteration of glycan residues structure may play a crucial role in investigating the mechanism and progression of arsenical skin cancers. Finally, WPK-FPA-FTIR imaging might serve as a potential and economic platform to aid in disease diagnosis of abnormal glycosylation-related diseases as compared with antibody-based assays.

## Figures and Tables

**Table 1:** Peak assignment of FTIR spectra of skin cell samples.

KC	HaCaT	HSC-1	Assignment
Wavenumbers/cm^−1^			
3294	3295	3295	Amide A (N-H stretching vibration)
3063	3062	3064	Amide B (overtone of amide II)
2958	2957	2957	ν_as_CH_3_ (CH_3_ antisymmetric stretching vibration, dominant contribution from proteins)
2924	2923	2923	ν_as_CH_2_ (CH_2_ antisymmetric stretching vibration, dominant contribution from lipids)
2872	2873	2872	ν_s_CH_3_ (CH_3_ symmetric stretching vibration, dominant contribution from proteins)
2853	2853	2852	ν_s_CH_2_ (CH_2_ symmetric stretching vibration, dominant contribution from lipids)
1738	1738	1738	ν_s_C=O (acid esters)
1650	1649	1657	Amide I (C=O stretching vibration, proteins)
1542	1542	1544	Amide II (vibration motion coupled C-N stretching vibration and C-N-H bending vibration)
1454	1454	1452	δ_as_CH_2_ (CH_2_ antisymmetric bending, lipids and proteins)
1390	1396	1389	δ_as_CH_3_ (CH_3_ antisymmetric bending, lipids and proteins)
1237	1238	1237	ν_as_PO^2−^ (PO^2−^ antisymmetric stretching vibration of DNA/RNA)
1080	1084	1080	ν_s_PO^2−^ (PO^2−^ symmetric stretching vibration of DNA/RNA)

## References

[1] Mead M.N. (2005). Arsenic: In search of an antidote to a global poison. Environ. Health Perspect..

[2] Chen C.J., Hsu L.I., Wang C.H., Shih W.L., Hsu Y.H., Tseng M.P., Lin Y.C., Chou W.L., Chen C.Y., Lee C.Y. (2005). Biomarkers of exposure, effect, and susceptibility of arsenic-induced health hazards in Taiwan. Toxicol. Appl. Pharmacol..

[3] Yu H.S., Lee C.H., Jee S.H., Ho C.K., Guo Y.L. (2001). Environmental and occupational skin diseases in Taiwan. J. Dermatol..

[4] Tsai S.-M., Wang T.-N., Ko Y.-C. (1999). Mortality for certain diseases in areas with high levels of arsenic in drinking water. Arch. Environ. Health Int. J..

[5] Lee C.-H., Liao W.-T., Yu H.-S. (2010). Mechanisms and immune dysregulation in arsenic skin carcinogenesis. J. Cancer Ther..

[6] Kitchin K.T. (2001). Recent Advances in arsenic carcinogenesis: Modes of action, animal model systems, and methylated arsenic metabolites. Toxicol. Appl. Pharmacol..

[7] Kojima C., Ramirez D.C., Tokar E.J., Himeno S., Drobná Z., Stýblo M., Mason R.P., Waalkes M.P. (2009). Requirement of arsenic biomethylation for oxidative DNA damage. JNCI J. Natl. Cancer Inst..

[8] Lau A.T., Li M., Xie R., He Q.Y., Chiu J.F. (2004). Opposed arsenite-induced signaling pathways promote cell proliferation or apoptosis in cultured lung cells. Carcinogenesis.

[9] Wen G., Calaf G.M., Partridge M.A., Echiburu-Chau C., Zhao Y., Huang S., Chai Y., Li B., Hu B., Hei T.K. (2008). Neoplastic transformation of human small airway epithelial cells induced by arsenic. Mol. Med..

[10] Trouba K.J., Geisenhoffer K.M., Germolec D.R. (2002). Sodium arsenite-induced stress-related gene expression in normal human epidermal, HaCaT, and HEL30 keratinocytes. Environ. Health Perspect..

[11] Ma W., Zhao L., Yin K., Feng D., Yang F., Liang J., Chen H., Bi C., Li X., Wang Y. (2015). Effects of arsenic trioxide on proliferation, paracrine and migration of cardiac progenitor cells. Int. J. Cardiol..

[12] Singh K.P., Kumari R., Treas J., DuMond J.W. (2011). Chronic exposure to arsenic causes increased cell survival, DNA damage, and increased expression of mitochondrial transcription factor A (mtTFA) in human prostate epithelial cells. Chem. Res. Toxicol..

[13] Lee C.H., Wu S.B., Hong C.H., Liao W.T., Wu C.Y., Chen G.S., Wei Y.H., Yu H.S. (2011). Aberrant cell proliferation by enhanced mitochondrial biogenesis via mtTFA in arsenical skin cancers. Am. J. Pathol..

[14] Tuccillo F.M., de Laurentiis A., Palmieri C., Fiume G., Bonelli P., Borrelli A., Tassone P., Scala I., Buonaguro F.M., Quinto I. (2014). Aberrant glycosylation as biomarker for cancer: Focus on CD43. BioMed Res. Int..

[15] Granovsky M., Fata J., Pawling J., Muller W.J., Khokha R., Dennis J.W. (2000). Suppression of tumor growth and metastasis in Mgat5-deficient mice. Nat. Med..

[16] Burchell J., Poulsom R., Hanby A., Whitehouse C., Cooper L., Clausen H., Miles D., Taylor-Papadimitriou J. (1999). An α2,3 sialyltransferase (ST3Gal I) is elevated in primary breast carcinomas. Glycobiology.

[17] Chiricolo M., Malagolini N., Bonfiglioli S., Dall’Olio F. (2006). Phenotypic changes induced by expression of β-galactoside α2,6 sialyltransferase I in the human colon cancer cell line SW948. Glycobiology.

[18] Chen C.Y., Jan Y.H., Juan Y.H., Yang C.J., Huang M.S., Yu C.J., Yang P.C., Hsiao M., Hsu T.L., Wong C.H. (2013). Fucosyltransferase 8 as a functional regulator of nonsmall cell lung cancer. Proc. Natl. Acad. Sci. USA.

[19] Wei Y., Liu D., Ge Y., Zhou F., Xu J., Chen H., Yun X., Gu J., Jiang J. (2008). Down-regulation of β1,4GalT V at protein level contributes to arsenic trioxide-induced glioma cell apoptosis. Cancer Lett..

[20] Petibois C., Desbat B. (2010). Clinical application of FTIR imaging: New reasons for hope. Trends Biotechnol..

[21] Miller L.M., Bourassa M.W., Smith R.J. (2013). FTIR spectroscopic imaging of protein aggregation in living cells. Biochim. Biophys. Acta Biomembr..

[22] Nasse M.J., Walsh M.J., Mattson E.C., Reininger R., Kajdacsy-Balla A., Macias V., Bhargava R., Hirschmugl C.J. (2011). High-resolution Fourier-transform infrared chemical imaging with multiple synchrotron beams. Nat. Meth..

[23] Noreen R., Chien C.C., Chen H.H., Bobroff V., Moenner M., Javerzat S., Hwu Y., Petibois C. (2013). FTIR spectro-imaging of collagen scaffold formation during glioma tumor development. Anal. Bioanal. Chem..

[24] Krafft C., Shapoval L., Sobottka S.B., Geiger K.D., Schackert G., Salzer R. (2006). Identification of primary tumors of brain metastases by SIMCA classification of IR spectroscopic images. Biochim. Biophys. Acta Biomembr..

[25] Beljebbar A., Dukic S., Amharref N., Manfait M. (2010). Screening of biochemical/histological changes associated to C6 glioma tumor development by FTIR/PCA imaging. Analyst.

[26] Chiu L.F., Huang P.Y., Chiang W.F., Wong T.Y., Lin S.H., Lee Y.C., Shieh D.B. (2013). Oral cancer diagnostics based on infrared spectral markers and wax physisorption kinetics. Anal. Bioanal. Chem..

[27] Hsu M.M., Huang P.Y., Lee Y.C., Fang Y.C., Chan M.W., Lee C.I. (2014). FT-IR microspectrometry reveals the variation of membrane polarizability due to epigenomic effect on epithelial ovarian cancer. Int. J. Mol. Sci..

[28] Munro K.L., Bambery K.R., Carter E.A., Puskar L., Tobin M.J., Wood B.R., Dillon C.T. (2010). Synchrotron radiation infrared microspectroscopy of arsenic-induced changes to intracellular biomolecules in live leukemia cells. Vib. Spectrosc..

[29] Liao W.T., Chang K.L., Yu C.L., Chen G.S., Chang L.W., Yu H.S. (2004). Arsenic induces human keratinocyte apoptosis by the FAS/FAS ligand pathway, which correlates with alterations in nuclear factor-kappa B and activator protein-1 activity. J. Investig. Dermatol..

[30] Paul P.C., Chattopadhyay A., Manna A.K., Dutta S.K. (2004). Skin cancers in chronic arsenic toxicity—A study of predictive value of some proliferative markers. Indian J. Pathol. Microbiol..

[31] Huang Y., Zhang J., McHenry K.T., Kim M.M., Zeng W., Lopez-Pajares V., Dibble C.C., Mizgerd J.P., Yuan Z.M. (2008). Induction of cytoplasmic accumulation of p53: A mechanism for low levels of arsenic exposure to predispose cells for malignant transformation. Cancer Res..

[32] Sun X., Li B., Li X., Wang Y., Xu Y., Jin Y., Piao F., Sun G. (2006). Effects of sodium arsenite on catalase activity, gene and protein expression in HaCaT cells. Toxicol. In Vitro.

[33] Liao W.T., Lan C.C., Lee C.H., Yu H.S. (2011). Concentration-dependent cellular responses of arsenic in keratinocytes. Kaohsiung J. Med. Sci..

[34] Niewiarowska A., Caltabiano M.M., Bailey D.S., Poste G., Greig R.G. (1987). Alterations in lipid-linked oligosaccharide metabolism in human melanoma cells concomitant with induction of stress proteins. J. Biol. Chem..

[35] Shang J., Gao N., Kaufman R.J., Ron D., Harding H.P., Lehrman M.A. (2007). Translation attenuation by PERK balances ER glycoprotein synthesis with lipid-linked oligosaccharide flux. J. Cell Biol..

[36] Taniguchi N. (2007). A sugar-coated switch for cellular growth and arrest. Nat. Chem. Biol..

[37] Fu M., Qin C., Li W., Yan Y., Zeng L., Yang X. (2013). Effect of glucosamine and chitooligomer on the toxicity of arsenite against *Escherichia coli*. Carbohydr. Polym..

[38] Tian H., Zhuang G., Ma A., Jing C. (2012). Arsenic interception by cell wall of bacteria observed with surface-enhanced Raman scattering. J. Microbiol. Methods.

[39] Clarke B.R., Greenfield L.K., Bouwman C., Whitfield C. (2009). Coordination of polymerization, chain termination, and export in assembly of the *Escherichia coli* lipopolysaccharide O9a antigen in an ATP-binding cassette transporter-dependent pathway. J. Biol. Chem..

[40] Clarke B.R., Richards M.R., Greenfield L.K., Hou D., Lowary T.L., Whitfield C. (2011). *In vitro* reconstruction of the chain termination reaction in biosynthesis of the *Escherichia coli* O9a O-polysaccharide: The chain-length regulator, WbdD, catalyzes the addition of methyl phosphate to the non-reducing terminus of the growing glycan. J. Biol. Chem..

[41] Fujiwara Y., Yamamoto C., Hirooka T., Terada N., Satoh M., Kaji T. (2008). Arsenite but not arsenate inhibits general proteoglycan synthesis in cultured arterial smooth muscle cells. J. Toxicol. Sci..

[42] Fujiwara Y., Yamamoto C., Inagaki T., Satoh M., Kaji T. (2012). Bismuth protects against arsenite-induced inhibition of proteoglycan synthesis in cultured vascular endothelial cells. J. Toxicol. Sci..

[43] Lee C.H., Wu S.B., Hong C.H., Chen G.S., Wei Y.H., Yu H.S. (2013). Involvement of mtDNA damage elicited by oxidative stress in the arsenical skin cancers. J. Investig. Dermatol..

[44] Baker M.J., Trevisan J., Bassan P., Bhargava R., Butler H.J., Dorling K.M., Fielden P.R., Fogarty S.W., Fullwood N.J., Heys K.A. (2014). Using Fourier transform IR spectroscopy to analyze biological materials. Nat. Protoc..

[45] Kalmodia S., Parameswaran S., Yang W., Barrow C.J., Krishnakumar S. (2015). Attenuated total reflectance fourier transform infrared spectroscopy: An analytical technique to understand therapeutic responses at the molecular level. Sci. Rep..

[46] Press W.H., Teukolsky S.A., Vetterling W.T., Flannery B.P. (1992). Numerical Recipes in FORTRAN: The Art of Scientific Computing.

